# A System of Genito-Urinary Diseases, Syphilology and Dermatology

**Published:** 1893-10

**Authors:** 


### Book Notices.

A SYSTEM OF GENITO-URINARY DISEASES, SYPHILOLOGY AND DERMATOLOGY.—By various authors. Edited by Prince A. Morrow, A. M., M. D., clinical professor of Genito-Urinary Diseases, formerly lecturer on Dermatology, in the University of the City of New York, Surgeon to Charity Hospital, etc. In three volumes, profusely illustrated. Sold by subscription only. Price per volume, Cloth $6.50, Sheep $ ——, Half Russia $——. D. Appleton & Co., publishers, 1, 3 and 5 Bond St., New York. Volume 1, Genito-Urinary Diseases.

The first volume of this system is now ready and contains about 1100 pages, royal octavo.

It is quite the fashion of late years to write books in the form of systems. An editor has general supervision of the work, arranges each individual subject, selects one or more authors for each, etc. In this way a division of labor and co-operation of thought is secured, and the result is a book far more thorough and complete than if written entirely by one person.

In his work as editor of this volume, Dr. Morrow has been most fortunate in his selection of authors, and we cannot too highly commend the result of their combined efforts. The following is a list of the subjects and authors:

Anatomy and Physiology of the Genito-Urinary Organs, by George Woolsey, M. D., New York; Diseases of the Penis, by Ramon Guiteras, M. D., New York; Diseases and Injuries of the Urethra, by F. Tilden Brown, M. D., New York; Etiology of Urethritis, by S. Lustgarten, M. D., New York; Acute Urethriti-Gonorrhœa, by George Emerson Brewer, M. D., New York; Chronic Gonorrhœa or Gleet, by William K. Otis, M. D., New York; Endoscopy, by Herman G. Klotz, M. D., New York; Gonorrhæal Ophthalmia, by Joseph A. Andrews, M. D., New York; Gonorrhœal Rheumatism, by Frank Harley, M. D., New York; Genorrhœa of Rectum, Nose, Mouth, Ear, Umbilicus and Axilla, by James P. Tuttle, M. D., New York; Stricture of the Urethra, by J. William White, M. D., Philadelphia; Diseases of the Prostate, by W. T. Belfield, M. D., Chicago; The Functional Disorders of Micturition, by Joseph D. Bryant, M. D., New York; Diagnostic Significance of Pathological Modifications of the Urine (including the most practical methods of urine analysis), by Eugene Fuller, M. D., New York; Urinary Fever, by J. A. Fordyce, M. D., New York; Cystoscopy, by

Willy Meyer, M. D., New York; The Cystites, by Samuel Alexander, M. D., New York; Injuries and Diseases of the Bladder, by George Ryerson Fowler, M. D., New York; Rnpture of the Bladder, by Alexander M. Stein, M. D., New York; Tumors of the Bladder, by Francis Sedgwick Watson, M. D., Boston; Stone in the Bladder, Prostate Urethra and Ureters, by Arthur T. Cobot, M. D., Boston; The Surgical Diseases of the Kidney, by Lewis A. Stinson, M. D., New York; Tuberculosis Uro-Genitalis, by John P. Bryson, M. D., St. Louis; Diseases of the Scrotum, by Charles W. Allen, M. D., New York; Diseases of the Testicle, by Jones Bell, M. D., Montreal, Canada; Diseases of the Testicle, by Edwin C. Burnett, M. D., St. Louis; Hydrocele and Spermatocele, by John A. Wyeth, M. D., and W. W. Van Arsdale, M. D., New York; Varicocele, by Edward L. Keys, M. D., New York; New Diseases of the Seminal Vesicles, by Paul Thorndike, M. D., Boston; Functional Disorders of the Male Sexual Organs, by Prince A. Morrow, M. D., New York; Gonorrhæa in the Female, by Andrew F. Currien, M. D., New York. After presenting the above list comment can be hardly considered as necessary.

It will be observed that a number of subjects not ordinarily found in text-books on Genito-Urinary diseases are discussed here, such as the chapters on functional disorders of micturition and their relation to various morbid states, the diagnostic significance of pathological modifications of the urine, urine analysis, etc. The chapters on endoscopy and cystoscopy contain elaborate and exhaustive presentations of the latest knowledge respecting these valuable aids to diagnosis and treatment.

It is a characteristic feature of the entire work to go into careful details in considering the subjects of diagnosis and treatment, and to present them in a clear and practical light.

In summing up the contents and merits of the book, it is evident from the many important additions that have been made to our knowledge, of the subjects they embrace, that a new and standard work of this kind was demanded, that the edition has selected only distinguished specialists as authors, each one of whom was selected for his special fitness to write on the subject assigned him—that each of these has performed his work in a thoroughly practical way, from his own independent stand point, that it is free from repetitions, though written by about thirty different authors, and that the work is sufficiently comprehensive to serve as a compendium of reference.

The editor and authors are to be congratulated on bringing out a book of so much genuine worth to the general practitioner as well as the specialist.

The book is profusely illustrated with chromo-lithographs and engravings. The type is large and clear, and the general make up of the book is excellent. H.

